# Bismuth/Porous Graphene Heterostructures for Ultrasensitive Detection of Cd (II)

**DOI:** 10.3390/ma13225102

**Published:** 2020-11-12

**Authors:** Luyi Huang, Yoshikazu Ito, Takeshi Fujita, Xingbo Ge, Ling Zhang, Heping Zeng

**Affiliations:** 1 School of Optical-Electrical and Computer Engineering, University of Shanghai for Science and Technology, Shanghai 200093, China; 18917095979@163.com (L.H.); hpzeng@phy.ecnu.edu.cn (H.Z.); 2 Institute of Applied Physics, Graduate School of Pure and Applied Sciences, University of Tsukuba, Tennodai, Tsukuba 305-8571, Japan; ito.yoshikazu.ga@u.tsukuba.ac.jp; 3 School of Environmental Science and Engineering, Kochi University of Technology, 185 Miyanokuchi, Tosayamada, Kami City, Kochi 782-8502, Japan; fujita.takeshi@kochi-tech.ac.jp; 4 The Center of New Energy Materials and Technology, School of Chemistry and Chemical Engineering, Southwest Petroleum University, Chengdu 610500, China

**Keywords:** porous materials, graphene, bismuth, electrochemical deposition, square wave anodic stripping voltammetry

## Abstract

Heavy metals pollution is one of the key problems of environment protection. Electrochemical methods, particularly anodic stripping voltammetry, have been proven a powerful tool for rapid detection of heavy metal ions. In the present work, a bismuth modified porous graphene (Bi@PG) electrode as an electrochemical sensor was adopted for the detection of heavy metal Cd^2+^ in an aqueous solution. Combining excellent electronic properties in sensitivity, peak resolution, and high hydrogen over-potential of bi-continuous porous Bi with the large surface-area and high conductivity on PG, the Bi@PG electrode exhibited excellent sensing ability. The square wave anodic stripping voltammetry response showed a perfect liner range of 10^−9^–10^−8^ M with a correlation coefficient of 0.9969. The limit of detection (LOD) and the limit of quantitation (LOQ) are calculated to be 0.1 and 0.34 nM with a sensitivity of 19.05 μA·nM^−1^, which is relatively excellent compared to other carbon-based electrodes. Meanwhile, the Bi@PG electrode showed tremendous potential in composite detection of multifold heavy metals (such as Pb^2+^ and Cd^2+^) and wider linear range.

## 1. Introduction

Heavy metals sensing is important to protect both humans and animals from damage of its toxicity and to keep our environment safe. Several methods have been developed for detecting and monitoring heavy metal ions (environment pollutants), such as atomic absorption spectroscopy (AAS) [1], atomic fluorescence spectrometry (AFS), and inductively coupled plasma mass spectrometry (ICP-MS) [2], and most of them require large size instruments and complex pretreatments for precise quantity analysis (i.e., skilled personnel). In contrast, the electrochemical technique is simpler to operate and easier for on-site analysis, and has been considered an efficient and portable method to detect heavy metal ions [3].

Electrochemical sensing, which has been extensively applied in environmental and industrial monitoring, can detect target analytes through a catalytic or binding event at the interface of electrodes [4,5,6,7]. The performance of the electrochemical sensor is determined by the surface area for molecular adsorption and conductivity of the electrode for sensitivity. Bismuth modified electrodes show excellent peak resolution with high reproducibility, high over-potential of hydrogen evolution, and wide operational window [8,9], promising a good candidate in the electroanalysis field owning to their low toxicity. In order to improve the sensitivity [10], several approaches have been proposed to fabricate functional electrode materials. In particular, bismuth nanoparticles deposited electrodes exhibit highly sensitive and reproducible performance for trace analysis of heavy metal ions with anodic stripping voltammetry approaches [6]. The formation of multicomponent alloys makes a great contribution to the excellent behavior of bismuth modified electrodes, likewise the association of the great properties of nanostructured material owes a great improvement to sensibility [11].

Carbon materials, especially graphene and its derivatives, have been widely investigated due to their diversity, favorable features, and active applications including electrochemical analysis [12,13]. Three-dimensional (3D) nanoporous graphene, which preserved the distinct 2D coherent electronic properties such as high conductivity (~10^4^ S/m) and electron mobility (5000–7000 cm^2^/Vs) [14,15], is expected to be a good electrode supporter. Meanwhile, bicontinuous open porous structure with high porosity (99.9%) and large surface area (1000 m^2^/g) become a good platform for electrochemical applications [16]. Besides, the high chemical stability of graphene ensures stable electrode performance and long lifetime [17]. Here, we constructed bismuth/nanoporous graphene heterostructures by depositing bismuth on 3D nanoporous graphene which serves as a template with a potentiostatic method. We synthesized bismuth covered 3D nanoporous graphene (Bi@PG) and applied to detect heavy metal ions via square wave anodic stripping voltammetry (SWASV). The Bi@PG serving as the working electrode showed excellent performance of 0.1 nM (namely 11.2 ppb) detection limit of Cd ions and relatively high sensitivity of 19 μA·nM^−1^. In addition, this electrode also kept high sensitivity and repeatability for Cd ions detection even in interfering ions (Zn^2+^, Na^+^, K^+^, Ca^2+^, and Cl^−^).

## 2. Result and Discussion

### 2.1. Synthesis and Characterization of Bi@PG

**Figure 1:**
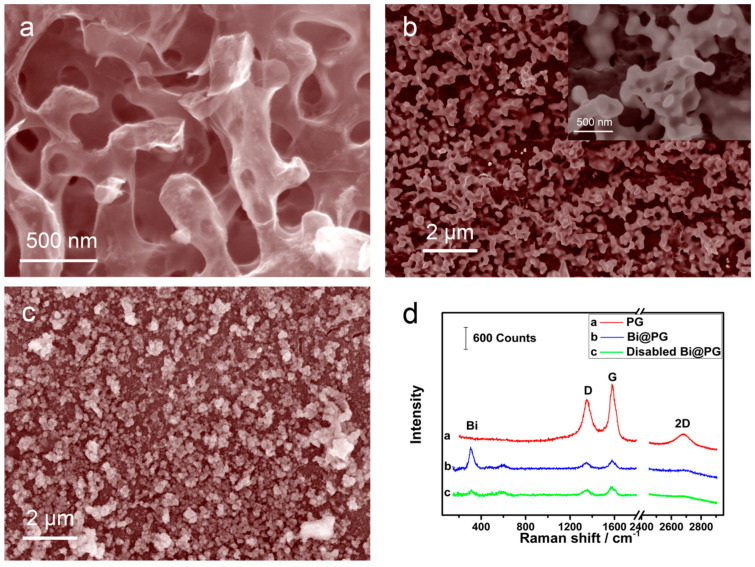
SEM images of (**a**) porous graphene (PG), (**b**) Bi@PG and (**c**) disabled Bi@PG; (**d**) Raman spectra of PG, Bi@PG, and disabled Bi@PG; the wavelength of excitation of the laser is 532 nm and the power is 10 mW.

Bismuth were deposited on nanoporous graphene [16] in static 0.2 M H_2_SO_4_ electrolyte consisting of 2 mM Bi^3+^ at −0.3 V (vs. Ag/AgCl) to form Bi@PG via a conventional electrochemical plating method. The SEM micrographs and the Raman spectra of PG and Bi@PG were shown in Figure 1. The interconnected nanoporous structure of the 3D graphene is uniform with an average pore size of ~210 nm (see Figure 1a). After bismuth depositing, the bismuth thin layer, together with localized clusters, uniformly distribute along the tubular bicontinuous graphene ligaments and form a new bismuth skeleton (Figure 1b), and the structure was partly degraded after cycling test (Figure 1c). The corresponding Raman spectra (Figure 1d) indicates that the PG was few layer graphene because of the 2D band at 2700 cm^−1^, and the appearance of the D band at 1350 cm^−1^ indicates the existence of structural defects in 3D porous graphene [16]. After bismuth deposition, two Raman peaks existed at 305 and 449 cm^−1^, which are consistent with α-Bi_2_O_3_ that forms naturally on the bismuth metal surface [18]. Meanwhile, the Raman peaks attributed to PG become weaker, onfirming a thin layer of bismuth was covered on the ligament surface of PG. With the cycling test, the intensities of the peaks belonging to α-Bi_2_O_3_ dramatically deceased while the intensity of PG seldom changed, indicating the bismuth layer was destroyed during ions detection. However, signal intensities of Raman peaks belonging to graphene’s D band, G band, and 2D band remain unchanged, further proofs that the graphene skeleton was stable, and electrochemical test partly destroyed the bismuth layer, leading to the electrode degradation.

**Figure 2:**
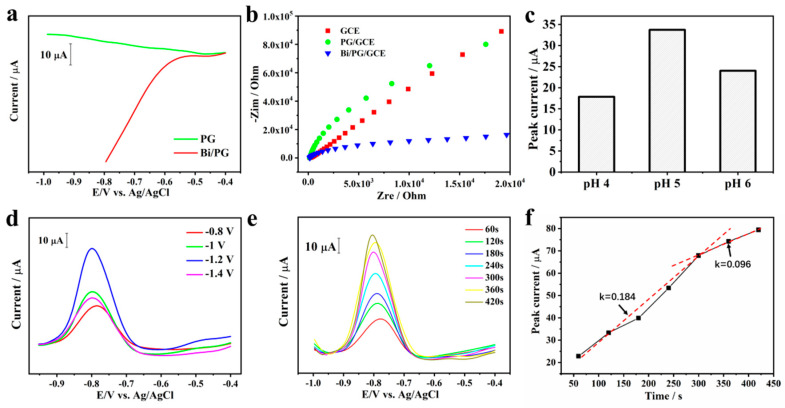
(**a**) SWASV of PG and Bi@PG in 0.2 M (pH 5.0) acetic acid buffer solution; (**b**) Nyquist plots of GCE, PG/GCE, and Bi@PG/GCE in 0.1 M NaCl solution; (**c**) effect of pH values on the peak current responses; (**d**) SWASV of Bi@PG in 10^−9^ M Cd^2+^/0.2 M (pH5.0) acetic acid buffer solution with different deposition potential; (**e**) SWASV of Bi@PG in 10^−9^ M Cd^2+^/0.2 M (pH 5.0) acetic acid buffer solution with different deposition time; (**f**) effect of Cd^2+^ deposition time on the peak current responses. Note: k is the slope value, and estimated by k = ΔI_current_/Δt.

For an electrode sensing with the SWASV method, the width of the working potential window and window anodic potential are important. Thus, we firstly checked the working potential window of PG before and after Bi deposition in pH5.0 acetic acid buffer solution. As shown in Figure 2a, the SWASV line on the PG electrode starts declining from −0.6 V due to hydrogen evolution, while the SWASV line on the Bi@PG electrode showed almost straight from −1.0 to −0.4 V and did not appear an apparent influence of hydrogen evolution currents until −1.0 V. That proves modification of bismuth nanoparticles significantly improves the stability of PG among the negative over-potential.

The electrochemical performance of the Bi@PG electrode was further evaluated using electrochemical impedance spectroscopy. We fixed PG and Bi@PG on glass carbon electrodes (GCE) with 0.05 wt.% Nafion. The impedance was measured at a potential of 150 mV with 5 mV perturbation voltage (vs. Ag/AgCl). Figure 2b showed the Nyquist plots of GCE, PG/GCE, Bi@PG/GCE in the 0.1 M NaCl solution at the frequency range from 10^−2^ to 10^5^ Hz, and the electron transfer ability of the electrode relates to the semicircle diameter. As shown in the figure, PG/GCE showing a relatively larger semicircle compared with that of Bi@PG/GCE indicates that the charge transfer resistance of Bi@PG/GCE decreased compared with PG/GCE, suggesting the presence of the Bi layer makes the electron transfer easier. Based on the experiments above, Bi@PG shows better electrochemical performance with a wide working window and accelerated electron transfer kinetics.

### 2.2. Optimization of Sensing Conditions

We optimized various voltammetric parameters of SWASV, including deposition potential, pH value, and deposition time, with the purpose of getting a better current curve for Cd^2+^ simultaneous detection on Bi@PG.

Deposition potential. We studied the impact of deposition potentials on the SWASV curve of Cd^2+^ with 300 s accumulation at the potentials from −0.8 to −1.4 V in 0.2 M acetate buffer solution at pH = 5.0. As shown in Figure 2d, with the deposition potential shifting from −0.8 to −1.2 V, the Cd^2+^ stripping peak currents increase. Whereas the stripping peak currents reduce when the accumulation potential is negative than −1.2 V, which may be caused by the hydrogen generation on the surface of the electrode under such negative potential [19]. The bubbles aggregated by hydrogen might block the Cd^2+^ deposition on the electrode surface, resulting in a decrease in current intensity at such negative potential [20]. Hence, −1.2 V was selected as the deposition potential for further measurement.

pH value. As it is known that pH value also affects the stripping peak current, thus we investigated the effect of pH value in 0.2 M NaAc–HAc buffer solution (ABS) with various pH value after Cd^2+^ accumulating 300 s at the potential −1.2 V. As shown in Figure 2c, the peak current for Cd^2+^, which was generated by Cd stripping during SWASV, intuitively varies with the pH value, and reaches a maximum value at pH 5.0. From pH = 4.0 to pH = 5.0, both electrostatic attraction and fully ionization of metal into cationic form are contributed to the peak current increase. As for the decrease of the stripping signal for Cd^2+^ from pH = 5.0 to pH = 6.0, it may be related to the hydrolysis of metal ions [15]. Hence, 5.0 was chosen as the proper pH value in 0.2 M ABS.

Deposition time. To further optimize deposition time, various accumulation times ranged from 50 to 450 s were investigated in 10^−7^ M Cd^2+^ solution. Figure 2f,e depict that the response of the stripping peak currents of Cd^2+^ increases with the deposition time increasing. For the deposition time from 50 to 300 s, a linear relation with a larger slope of k = ΔI_current_/Δt ≈ 0.184 is obtained, and the slope decreases to 0.096 from 300 to 450 s. The variation of the slopes implies that the metal ions accumulation speed is not constant and is related to the surface condition of the electrode. With the accumulation of Cd^2+^, the electrode was covered by Cd metal and which will influence the loading speed. Therefore, 300 s was chosen as the deposition time for subsequent tests. If we decrease the concentration of Cd^2+^, the best accumulation time will increase (see Figure 2e).

### 2.3. Stripping Behavior toward Cd^2+^

**Figure 3:**
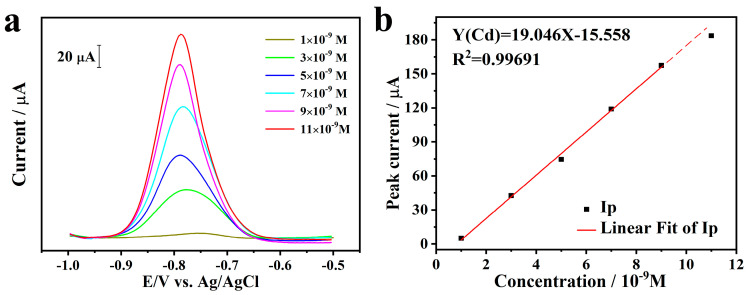
(**a**) SWASV of Bi@PG in 0.2 M (pH = 5.0) acetic acid buffer solution with gradient concentration of Cd^2+^; (**b**) The corresponding linear calibration plots of peak currents vs. concentrations of Cd^2+^.

Under the optimal experimental conditions, the simultaneous determination of Cd^2+^ was performed on Bi@PG using SWASV. Figure 3a shows the SWASV responses for Cd^2+^ at different concentrations, and the detection limit is around 1 nM with 300 s deposition time. Well-defined peaks of Cd^2+^ are obtained with a maximum current at the potential of −0.78 V. Figure 3b shows the corresponding linear calibration plots of stripping peak currents against the concentrations of Cd^2+^. It can be seen that the stripping response for Cd^2+^ concentration exhibits good linearity. The linearization equation for Cd^2+^ is y = 19.046x − 15.558 with a correlation coefficient of 0.9969 from 1 to 9 nM, where x is the concentration with the unit of nM and y represents current with the unit of μA. The limit of detection (LOD) and the limit of quantitation (LOQ) are calculated to be 0.1 and 0.34 nM based on three times background noise and ten times background noise, respectively. When the concentration of Cd^2+^ reaches more than 11 nM, the peak current slightly increases, and the linear relationship between current intensity and liquid concentration on longer exist due to the electrode becomes saturated with heavy metal ions which has been observed before [21].

For comparation, simultaneous determination of Cd^2+^ with other electrodes based on carbon materials and bismuth were summarized in Table 1. It can be found that the analytical performance of Bi@PG electrodes possesses superior LOD and sensitivity during a relatively smaller linear range.

### 2.4. Test of Repeatability and Anti-Interference Ability

**Figure 4:**
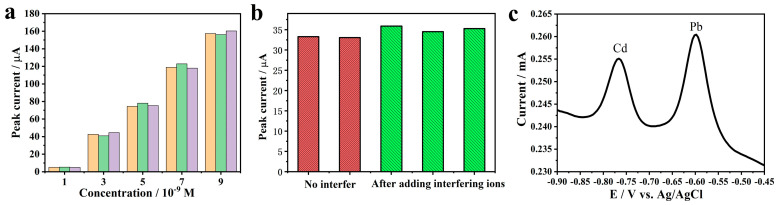
(**a**) The repeatability of Bi@PG for repetitive measurements via SWASV; (**b**) Effect of interfering ions (such as Zn^2+^, Na^+^, K^+^, Ca^2+^, and Cl^−^) on Cd^2+^ detection; (**c**) SWASV of Bi@PG in 0.2 M (pH = 5.0) acetic acid buffer solution with 10^−7^ M Cd^2+^ and Pb^2+^.

We investigated the repeatability of Bi@PG by reusing the same sample for repetitive measurements. As shown in Figure 4a, the stripping currents appears an average relative standard deviation (RSD) of 2.6% and an average standard deviation (SD) of 1.72 for gradient concentration Cd^2+^ cycle detection. Meanwhile, we calculated the corresponding concentration using the linearization equation in the preceding part and got the standard uncertainty which is 0.2 nM on average. Additionally, we added 100 times concentrated interfering ions such as Zn^2+^, Na^+^, K^+^, Ca^2+^, and Cl^−^ into the ABS buffer, and the results are shown in Figure 4b. It can be seen that the characteristic peak currents increase by 6.2% averagely and still exist a RSD of 4.2% for Cd^2+^. Though the basic current was improved, adding interfering ions did not influence the peak currents seriously. According to the above, this Bi@PG electrode showed favorable repeatability and anti-interference ability.

SWASV studies were also conducted with solution containing Pb^2+^ and Cd^2+^ at 10^−7^ M concentrations. The separation of peak potentials for the two metals, as shown in Figure 4c, is a preliminary indication of the ability of Bi@PG to detect multifold types of metals simultaneously.

## 3. Conclusions

We systematically investigated the electrochemical determination of Cd^2+^ using bismuth nanoparticles deposited 3D nanoporous graphene electrode. The Bi@PG hybrid electrode appears good sensitivity for Cd^2+^ with a detection limit of 0.1 and 0.34 nM quantitation limit. Meanwhile, the hybrid electrode exhibits excellent anti-interference ability and good reproducible ability. In addition, free-standing and bicontinuous 3D nanoporous graphene with the large surface area and open porosity provide ideal support to the bismuth nanoparticles. Thus, the hybrids as portable electrochemical sensors could develop utilization of detecting other metal ions and enhance the sensitivity in practical conditions containing interfering ions.

## 4. Experimental Sections

### 4.1. Material

Porous graphene was synthesized with the Ni framework [16]. Analytical grade ZnCl_2_, NaCl, KCl, CaCl_2_, HCl, and H_2_SO_4_ were obtained from Sinopharm Chemical Reagent (SCR, Shanghai, China) and used as received. Analytical grade Pb(NO_3_)_2_ and NaAc were obtained from Aladdin Industrial Corporation (Shanghai, China) and used as received. Analytical grade Bi(NO_3_)_3_·5H_2_O was obtained from Sigma Aldrich (St. Louis, MO, USA) and used as received. Ultrapure water with resistivity of 18.2 MΩ was produced by machine from Chengdu YouPu Biotechnology Co. Ltd., (Sichuan, China).

### 4.2. Instruments

Electrochemical measurement was carried out with the three electrodes system (Shanghai Chen hua, CHI650E), and microstructures of as-prepared porous graphene and Bi@PG were obtained by a Quanta FEG 250 scanning electron microscope (SEM) (ThermoFisher Scientific, Waltham, MA, USA).

### 4.3. Electrodes Preparation and Modification

Potentiostatic deposition was used to depositing Bi on the surface of PG with a constant potential of −0.3 V for 10 min in the solution of 0.2 M H_2_SO_4_ with 2 mM Bi^3+^.

### 4.4. Electrochemical Detection of Heavy Ions

SWASV was used for the observation of electrochemical behavior under the optimized conditions. Cd was deposited at a constant potential of 1.2 V for 300 s by the reduction of Cd^2+^ in 0.1 M HAc–NaAc (pH = 5.0) acid solution. The anodic stripping of the electrodeposited metal was performed in the potential range from−1.0 to 0.4 V. Prior to the next cycle, and a desorption potential of −0.4 V for 300 s was applied to remove the residual metals under stirring conditions.

## Figures and Tables

**Table 1:** Comparison of different electrodes based on Bi or carbon material for the determination of Cd^2+^.

Electrode	Linear Range	Detection Limit	Sensitivity	Method	Ref.
RGO/Bi/GCE	20–120 μg/L	2.8 μg/L	1.25 μA·μg^−1^·L	SWASV	[22]
CNTs/Bi	20–100 μg/L	0.7 μg/L	1.45 μA·μg^−1^·L	SWASV	[23]
Bi/CPE	10–100 μg/L	1.2 μg/L	N.M.	ASV	[24]
GR/BiF/Nafion/IL/SPCE	0.1–100 μg/L	0.06 ng/L	0.52 μA·μg^−1^·L	SWASV	[25]
Bi_2_O_3_/CNTs	1.5–20 μg/L	0.22 μg/L	1.55 μA·μg^−^^1^·L	SWASV	[26]
Hg-Bi/PDAAQ/GC	0–50 μg/L	0.107 μg/L	3.763 μA·μg^−^^1^·L	SWV	[27]
L-cystine-rGO/GCE	44.9–225 μg/L	0.366 μg/L	24.5 nA·μg^−1^·L	DPASV	[28]
CNTs tower	112–449 μg/L	2.8 μg/L	7.5 nA·μg^−1^·L	ASV	[29]
MWCNTs/NA/Bi/SPE	0.5–80 μg/L	0.1 μg/L	13.42 mA·μg^−1^·L	DPASV	[30]
TiO_2_/GR	67.2–3584 μg/L	0.22 μg/L	17.3 μA·μg^−1^·L	DPASV	[31]
Bi@PG	0.11–1.12 μg/L	0.011 μg/L	0.17 mA·μg^−1^·L	SWASV	This work

(ASV: Anodic stripping voltammetry, DPASV: Differential pulse anodic stripping voltammetry, RGO: Reduced graphene oxide, CNT: Carbon nanotube, CPE: Carbon paste electrode, GR: Graphene, IL: Ionic liquid, SPCE: Screen printed carbon electrode, PDAAQ: poly(1,2-diaminoanthraquinone), MWCNTs: multiwalled carbon nanotubes, NA: Nafion, N.M.: Not mentioned).
